# Transcriptome and Regulatory Network Analyses of CD19-CAR-T Immunotherapy for B-ALL

**DOI:** 10.1016/j.gpb.2018.12.008

**Published:** 2019-06-13

**Authors:** Qiong Zhang, Hui Hu, Si-Yi Chen, Chun-Jie Liu, Fei-Fei Hu, Jianming Yu, Yaohui Wu, An-Yuan Guo

**Affiliations:** 1 Hubei Bioinformatics and Molecular Imaging Key Laboratory, Department of Bioinformatics and Systems Biology, Key Laboratory of Molecular Biophysics of the Ministry of Education, College of Life Science and Technology, Huazhong University of Science and Technology, Wuhan 430074, China; 2 Institute of Hematology, Union Hospital, Tongji Medical College, Huazhong University of Science and Technology, Wuhan 430022, China

**Keywords:** CAR-T, B-ALL, Transcriptome profile, lncRNA, Regulatory network

## Abstract

Chimeric antigen receptor (CAR) T cell therapy has exhibited dramatic anti-tumor efficacy in clinical trials. In this study, we reported the **transcriptome profiles** of bone marrow cells in four B cell acute lymphoblastic leukemia (**B-ALL**) patients before and after CD19-specific **CAR-T** therapy. CD19-CAR-T therapy remarkably reduced the number of leukemia cells, and three patients achieved bone marrow remission (minimal residual disease negative). The efficacy of CD19-CAR-T therapy on B-ALL was positively correlated with the abundance of CAR and immune cell subpopulations, *e.g.*, CD8^+^ T cells and natural killer (NK) cells, in the bone marrow. Additionally, CD19-CAR-T therapy mainly influenced the expression of genes linked to cell cycle and immune response pathways, including the NK cell mediated cytotoxicity and NOD-like receptor signaling pathways. The **regulatory network** analyses revealed that microRNAs (*e.g.*, miR-148a-3p and miR-375), acting as oncogenes or tumor suppressors, could regulate the crosstalk between the genes encoding transcription factors (TFs; *e.g.*, *JUN* and *FOS*) and histones (*e.g.*, *HIST1H4A* and *HIST2H4A*) involved in CD19-CAR-T therapy. Furthermore, many long non-coding RNAs showed a high degree of co-expression with TFs or histones (*e.g.*, *FOS* and *HIST1H4B*) and were associated with immune processes. These transcriptome analyses provided important clues for further understanding the gene expression and related mechanisms underlying the efficacy of CAR-T immunotherapy.

## Introduction

As one of the main types of leukemia, B cell acute lymphoblastic leukemia (B-ALL) is caused by the occurrence of genetic abnormalities in B cells, leading to the aberrant arrest of normal lymphoid maturation, evasion of apoptosis, and uncontrolled cell proliferation [1]. Given the limited success of chemotherapy and radiotherapy, the recently-emerging immunotherapy shows potent efficacy in treating cancers including B-ALL [2]. In particular, T cells with reprogrammed chimeric antigen receptors (CARs) for B cell malignancy-specific antigen CD19 (CD19-CAR-T) are considered to be a promising tool in the immunotherapy for B-ALL. CD19-CAR-T can recognize and eliminate tumor cells and has demonstrated remarkable efficacy on inducing remission in patients with relapsed/refractory B-ALL [3]. Notably, 70%–90% of patients with refractory B-ALL achieved a complete response (CR) at 2 weeks post CD19-CAR-T infusion [4]. Despite the side effects, such as cytokine release syndrome (CRS), neurologic toxicities, low blood cell counts, a weakened immune system, and even death [5], CAR-T therapy is regarded as a revolutionary treatment regimen for patients with advanced blood cancers, and thus one of the most successful immunotherapeutic approaches [6].

Most studies on CAR-T immunotherapy were focused on the clinical efficacy and new antigen development. However, few studies have investigated the alterations in gene expression and regulation of patients after CAR-T therapy. Transcriptome profiling has been widely used to investigate molecular mechanisms underlying the recurrence and therapy of cancers [7], [8], and to explore candidate target antigens for the improvement of immunotherapy efficacy [9]. Additionally, microRNAs (miRNAs) and transcription factors (TFs) represent two main regulators of gene expression [10], [11]. They could form regulatory modules and play critical roles in the development of immune cells [12] and tumorigenesis [13], thereby affecting immunotherapy [14]. Moreover, surveying the transcriptome profiling and regulatory networks of patients under different conditions (*e.g.*, remission or non-remission) could provide insights into the underlying molecular mechanisms involved in CAR-T therapy.

In this study, we investigated the clinical outcome and analyzed transcriptome profiles of bone marrow (BM) samples before and post CD19-CAR-T therapy from 4 adult patients with refractory B-ALL. Based on the analysis of differentially-expressed genes (DEGs), long non-coding RNAs (lncRNAs), and miRNAs, we proposed a schematic model of regulatory networks involved in the CD19-CAR-T therapy on B-ALL.

## Results

### Clinical information and outcomes in four cases with CD19-CAR-T therapy

**Figure 1:**
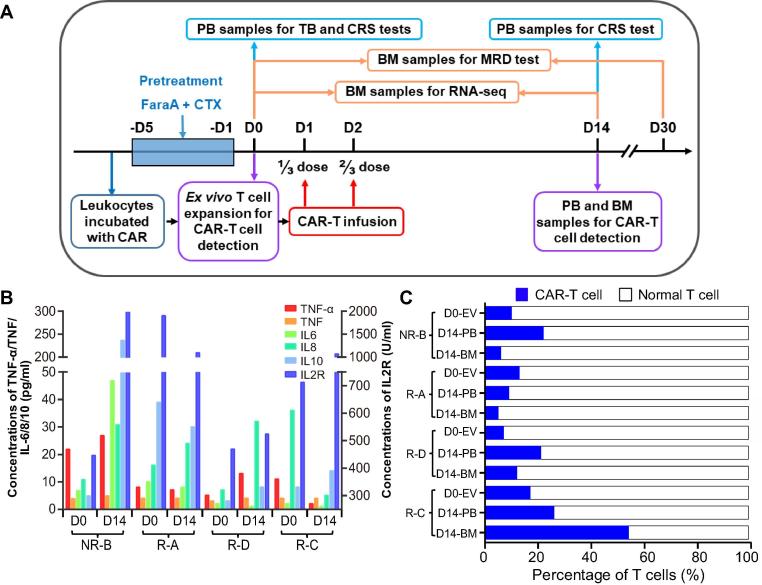
**The schedule of CD19-CART clinical trial and levels of CRS-related factors and CAR-T cells** **A.** The time course of CAR-T clinical trial and sampling arrangement for various examinations. The day before the CAR-T infusion was defined as D0. Patients were infused with CD19-CAR-T cells at 1/3 dose on D1 and 2/3 dose on D2, respectively. **B.** The levels of CRS-related cytokines in the serum. The scale for the concentrations of TNF, TNF-α, IL-6, IL-8, and IL10 is shown on the left, and the right Y axis shows the concentration for IL-2R. The four B-ALL patients were named A, B, C, and D, and the prefix of patients represent the effect of CAR-T therapy. **C.** The proportion of CAR-T cells. Percentage of CAR-T cells in CAR-T cell culture after *ex vivo* expansion (D0-EV) as well in the PB and BM samples collected from patients on D14 was determined using flow cytometry. B-ALL, B cell acute lymphoblastic leukemia; NR, non-remissive; R, remissive; CAR, chimeric antigen receptor; PB, peripheral blood; BM, bone marrow; CRS, cytokine release syndrome; FaraA, fludarabine; CTX, cetuximab.

Four patients approved for clinical trials of CD19-CAR-T therapy were selected in this study for further analysis (Table 1). The workflow of CAR-T therapy as well as clinical and biochemical examinations is shown in Figure 1A. The detailed procedures, including the construction of a 2nd generation CAR vector and CAR-T cell preparation, are presented in the Materials and methods section. After CD19-CAR-T infusion, the minimal residual disease (MRD) level was markedly decreased in all patients. Moreover, three out of the four patients, which were named as R-A, R-C, and R-D (Table 1), became MRD negative and achieved a molecular remission one month after CD19-CAR-T therapy. These results imply that the anti-tumor effects of CD19-CAR-T therapy played a profoundly positive role in the 4 patients, which was consistent with previous reports [15].

**Table 1:** **Clinical information of the four B-ALL patients for CAR-T therapy in this study**

**Patient**	**Gender**	**Age**	**Presence of *BCR*-*ABL* fusion gene**	**Pretreatment (mg/m2)**	**TB (%)** **D0**	**MRD** **D0**	**MRD** **D30**	**CRS grade** **D14**	**Relapse**
NR-B	Male	29	−	FaraA 30 + CTX 750	65.46	+	+	2	NR

R-A	Female	59	−	FaraA 30 + CTX 750	17.92	+	−	1	1.5 months

R-D	Male	53	+	FaraA 30	0.5	+	−	1	Remission and followed by transplantation

R-C	Female	52	−	FaraA 30	0.25	+	−	1	13 months

*Note*: The four B-ALL patients, A, B, C, and D, were classified to be R and NR, depending on their response to the CAR-T therapy. See Figure 1A for the time course of CAR-T therapy. “+” indicates the presence of MRD and “−” indicates the MRD levels below detection limit. B ALL, B cell acute lymphoblastic leukemia; CAR-T, chimeric antigen receptor T cell; NR, non-remissive; R, remissive; *BCR*-*ABL*, *BCR*-*ABL* fusion gene; FaraA, fludarabine; CTX, cetuximab; TB, tumor burden; MRD, minimal residual disease; CRS, cytokine release syndrome.

According to a previous report that the concentration of CAR-T cells reaches a peak *in vivo* at 2 weeks post infusion [16], we monitored alterations in the levels of cytokines and CAR-T cells on day 0 (D0, before CAR-T infusion) and day 14 (D14, 14 days after CAR-T infusion). The concentrations of IL-6/8/10/2R were dramatically increased after CAR-T infusion in the non-remissive (NR) patient (named as NR-B), suggesting a severe CRS (Figure 1B). The numbers of CAR-T cells were dramatically increased both in the peripheral blood (PB) and BM after CAR-T therapy in two patients and the proportion of CAR-T cells in PB was increased to 20% at D14 (Figure 1C). CAR-T cells accounted for 7.61%–17.74% of the CAR-T cell culture after *ex vivo* expansion (D0-EV, Figure 1A), whereas this ratio varied greatly in patients on D14 after CAR-T therapy, being 9%–27% in the PB and 5%–55% in the BM (Figure 1C). Notably, the ratio of CAR-T cells in the BM was twofold of that in the PB in patient R-C on D14. Coincidentally, the R-C patient with the highest ratio of CAR-T cells in both PB and BM remained in remission up to 13 months, whereas the patient NR-B with the lowest ratio of CAR-T cells did not achieve remission (Table 1).

### Transcriptome profile of BM from patients before and post CD19-CAR-T therapy

To investigate the transcriptional profiles of neoplastic nidus before and after CD19-CAR-T therapy, we performed RNA-seq and miRNA-seq analyses for BM samples from these 4 patients. As a result, we identified 10,263 genes and 470 miRNAs expressed in these samples with the threshold of fragments per kilobase of exon model per million reads mapped (FPKM) >1 for genes and transcripts per million reads mapped (TPM) >10 for miRNAs (Table S1). Basic statistics of sequencing data and gene expression profiles are presented in Table S2 and Figure S3, respectively. Among them, 85 protein-coding genes were highly expressed (FPKM >100) in all samples, whose functions were mainly associated with the structural constituent of ribosome and translation in the GO annotation (Figure S2). Meanwhile, expression of 5–10 miRNAs (such as let-7 family members) accounted for 70% of the entire miRNA expression abundance across the 4 patients (Figure S3B), suggesting their potential important regulatory roles in the BM. The partial least squares discriminant analysis (PLS-DA) shows that the transcriptome profiling of NR-B was different from those of other patients in remission (Figure S3C). In addition, according to the variable importance in projection (VIP) score, the top 20 genes contributing to the discrimination of all four samples shown in Figure S3C and histone genes stood out.

To investigate whether CAR-T therapy influenced the composition of T cell receptors (TCRs) in the neoplastic nidus, we examined the distribution of complementarity-determining region 3 (CDR3) sequences in RNA-seq data. A total of 685 CDR3 sequences were identified across samples. The R-D-D14 sample contained the highest number of CDR3 sequences, while the R-C-D14 sample had the lowest number. The number of CDR3 sequence varied among samples, and no dominant CDR3 sequence was found. The frequency of CDR3 sequences varied from 1% to 23% (Figure S3D), suggesting the absence of a dominant TCR clonotype in most of the samples before and after CAR-T therapy. These findings were consistent with a previous study, which shows that the CAR-based therapy may be independent from TCR signals or clone-specific events requiring antigen presentation and TCR recognition [17].

**Figure 2:**
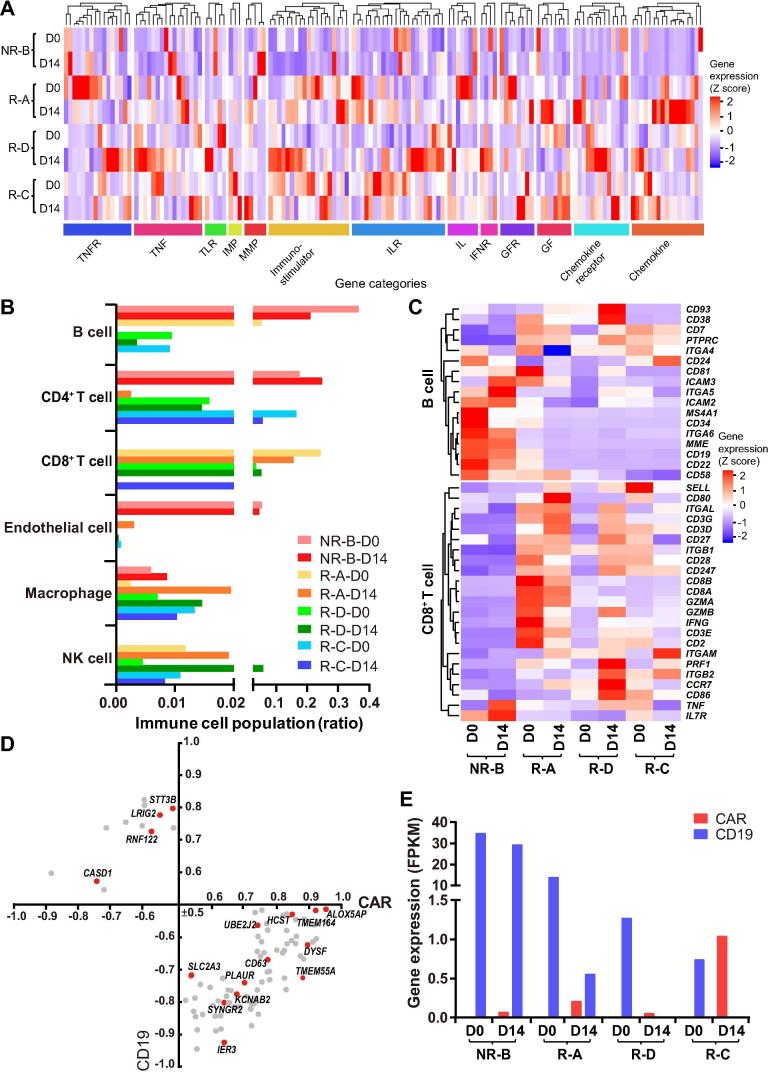
**Expression of CAR, CD19, and genes associated with immune functions in BM samples before and after CAR-T infusion** **A.** Heatmap of immune microenvironment and immunostimulator genes. The blue to red coloring in the legend indicates the gene expression level (Z score scaled) from low to high. Hierarchical clustering analysis was carried out by calculating the Euclidean distances. The categories of the microenvironment and immunostimulator genes determined by gene functions are indicated below the heatmap. **B.** The distribution of immune cell populations within the BM of patients among patients on D0 and D14. **C.** Heatmap of marker genes of B cells (including pro-B and pre-pro-B cells) and key genes related to the cytotoxic function of CD8^+^ T cell. **D.** Pearson's correlation of membrane protein genes for CD19 and CAR expression (*P* < 0.05, |correlation| ≥0.5). The red dot indicates the gene that was found to be involved in leukemia. **E.** The expression levels of the CAR and CD19 genes in the BM of patients on D0 and D14. All the data shown in this figure were based on RNA-seq analyses. TNF, tumor necrosis factor; TNFR, TNF receptor; TLR, toll like receptor; IMP, tissue inhibitor of metalloproteinase; MMP, matrix metallopeptidase; IL, interleukin; ILR, IL receptor; IFNR, interferon alpha and beta receptor; GF, growth factor; GFR, GF receptor; NK, natural killer.

The CAR-T therapy may lead to alterations in the tumor microenvironment and immune cell populations [18]. We found that the expression levels of microenvironment-related genes were markedly increased (fold change >2) in patients with a shorter remission time (R-A and R-D), in comparison with the best prognosis sample (R-C) (Figure 2A). In particular, expression of chemokines and immunostimulators was activated after CAR-T infusion in remissive patients, while these factors seemed not to respond to CAR-T therapy in the NR patient (Figure 2A, Table S3). The proportion of B cells and CD8^+^ T cells in the NR patient was notably different from the others (Figure 2B), suggesting a higher number of residual leukemic pre-B cells and a lower efficacy of CAR-T therapy in the NR patient. The expression levels of marker genes (*e.g.*, *CD19/CD10/CD22/CD34*) in leukemic pre-B cells were markedly decreased after CAR-T infusion in remissive patients (Figure 2C). Notably, compared to the patients in remission, T cells were rarely detected and the expression levels of genes involved in the activity of CD8^+^ T cells were much lower in the NR patient (Figure 2C).

The accessibility of antigen-presenting cells and the abundance of CAR-T cells could positively influence the effect of CAR-T immunotherapy on B-ALL [19], [20]. Thus, we examined the correlation between the CAR and *CD19* levels. As expected, the expression levels of CAR and *CD19* showed an opposite trend in D14 samples, which was consistent with the clinical outcomes (Figure 2E and Table 1). Meanwhile, we determined the correlation coefficients between the expressed membrane-protein genes and the relative abundances of *CD19* and CAR. As a result, we found that the expression levels of 89 membrane-protein genes were highly correlated with the CAR and/or *CD19* levels (Figure 2D), among which 16 have been reported to be associated with leukemia, such as *CD63*, a marker for malignant B cells [21].

### Differentially-expressed genes and functional modules relevant to the CD-19-CAR-T therapy

**Figure 3:**
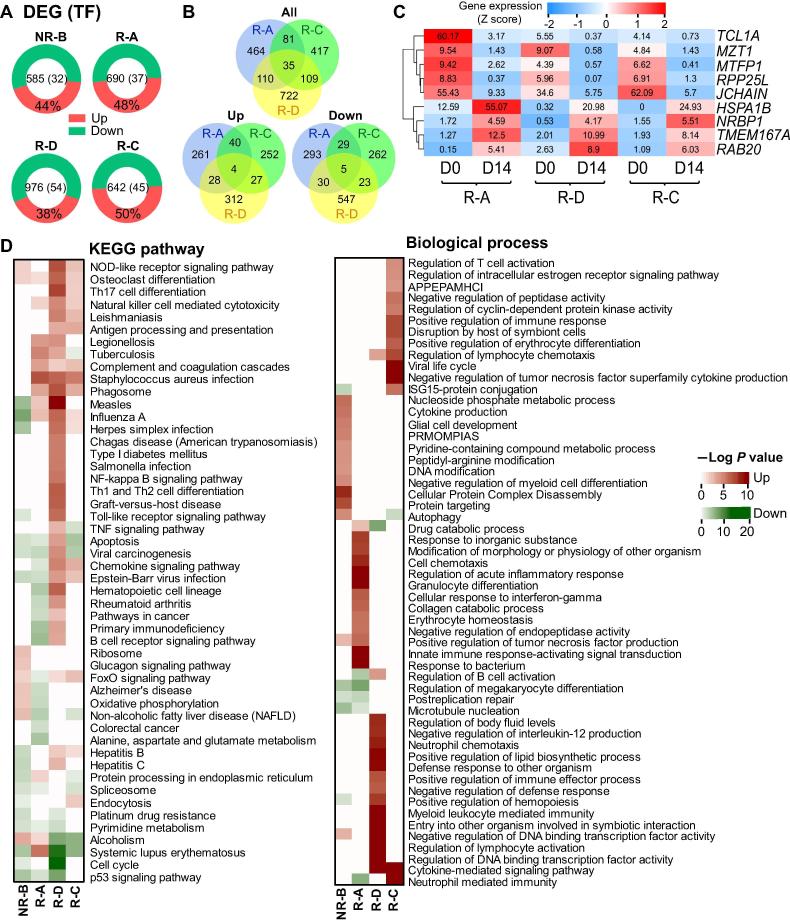
**The expression profile and functional enrichments of DEGs in BM samples after CAR-T infusion** **A.** The distribution of DEGs across the four patients with the percentage of the up-regulated DEGs provided. The number of total DEGs and those encoding TFs in each patient is presented in the center. **B.** Venn diagrams showing all (top), up-regulated (bottom left), and down-regulated (bottom right) DEGs that are common or specific across the three remissive samples, respectively. **C.** The heatmap demonstrating the common DEGs with the same expression tendency after CD19-CAR-T therapy in three remissive patients. The number in each cell indicates the expression value, while the color keys represent the expression tendency of genes after CD19-CAR-T therapy (blue: down-regulated, red: up-regulated). **D.** The heatmaps showing the enrichment of DEGs in terms of the KEGG pathways (left) and GO biological processes (right). The up-regulated DEGs enriched terms were shown in red, while the enriched results of down-regulated DEGs were in green. The *P* values were calculated using Fisher's exact test and log-transformed. “Up” and “Down” represent DEGs with up-regulated and down-regulated expression in D14 sample when compared to D0 sample in each patient, respectively. DEG, differentially-expressed gene; APPEPAMHCI, antigen processing and presentation of exogenous peptide antigen via MHC class I; PRMOMPIAS, positive regulation of mitochondrial outer membrane permeabilization involved in apoptotic signaling.

In total, we detected 585–976 differentially-expressed genes (DEGs) when comparing the gene expression levels before and after CD19-CAR-T infusion for each patient (Table S1). Eighty percent of DEGs were protein-coding genes, and ∼5% of which were TFs (Figure 3A). The highest number of DEGs was observed in the R-D patient carrying the *BCR*-*ABL* gene fusion. The three remissive patients shared 35 overlapping DEGs (Figure 3B), but only 9 genes showed the same expression trend (Figure 3B and C), implying their pivotal roles in the CAR-T therapy. For example, expression of *NRBP1*, the gene that encodes a tumor suppressor involved in cell death regulation, was up-regulated in remissive samples [22], while the expression of the poor prognosis indicators *JCHAIN* and *TCL1A* was both down-regulated [23], [24].

To further investigate biological functions of DEGs underlying CD19-CAR-T therapy, we performed the KEGG pathway and Gene Ontology (GO) enrichment analysis for all DEGs found in each patient. The top 20 enriched pathways and top 15 biological process terms are presented in Figure 3D. Although the enrichment results exhibited profound heterogeneity, most of the enriched terms were related to immune response and cell cycle. Notably, the up-regulated DEGs in the remissive patients were enriched in pathways including the natural killer (NK) cell mediated cytotoxicity and phagosome (Figure 3D). Consistent with the clinical outcome that the NR-B patient had a severe CRS and no remission (Figure 1B and Table 1), the up-regulated DEGs in the NR-B sample were mainly enriched in the cytokine-related terms and the acute inflammatory response processes (Figure 3D), while the down-regulated DEGs were enriched in the apoptosis and endocytosis related pathways. Interestingly, the osteoclast differentiation pathway was enriched in up-regulated DEGs for all patients after CAR-T therapy (Figure 3D).

### Co-expression and regulatory network analysis revealed the gene/lncRNA/miRNA modules involved in CAR-T therapy

**Figure 4:**
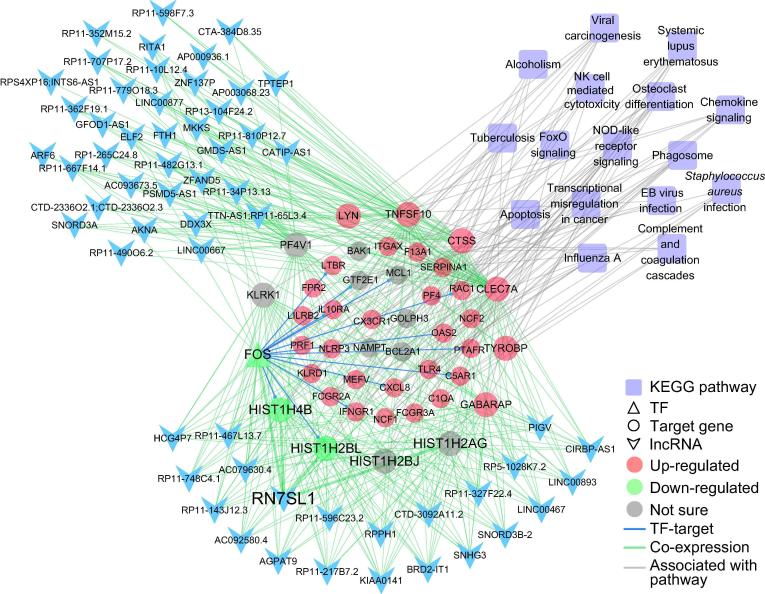
**TF–gene–lncRNA co-expression regulatory network containing the largest number of lncRNAs enriched in immune related pathways** Modules were detected by weighted correlation network analysis (WGCNA) using protein-coding genes and lncRNAs. The turquoise-colored module contains the largest number of lncRNAs and functionally enriched in the immune related pathways. TFs and their target gene(s) are indicated in triangles and circles, respectively. Genes up-regulated and down-regulated in all patients are indicated in red and green, respectively, whereas genes with an opposite expression tendency among patients are indicated with gray circles. lncRNAs are indicated with blue arrowheads and KEGG pathways are indicated with purple quadrangles. Edges indicating TF-target regulation, co-expression, and links between genes and pathways are shown in blue, green, and gray, respectively.

In addition to the functional enrichment analysis, we applied weighted gene correlation network analysis (WGCNA) to identify functional biological modules. In total, 18 co-expression modules were detected (Figure S4A), among which histone genes were significantly enriched (Chi-square test, *P* = 1.55E−9). Notably, 9 of 18 modules were highly correlated (*R* > 0.9, *P* < 1E−6) with the apoptosis, cell cycle, and immune-related pathways, such as the NK cell mediated cytotoxicity, NOD-like receptor signaling pathway, and phagosome terms (Figure S4B and Table S4). The turquoise-colored module contained a number of lncRNAs, and the protein-coding genes contained in this module were enriched in NK cell mediated cytotoxicity, phagosome, and NOD-like receptor signaling pathway (*P* values: 2.26E−4, 1.32E−4, and 4.7E−3, respectively) (Figure S4B and Table S4), which were markedly up-regulated after the CAR-T infusion (Figure 4 and Figure S4C). While the black-colored module containing fewer lncRNAs was mainly involved in the processes of cell cycle, HTLV-I infection, and Epstein–Barr (EB) virus infection (*P* values: 1.63E−11, 5.83E−5, and 1.65E−4, respectively) (Figure S4B and Table S4). These results suggested that the lncRNAs in these modules may be involved in the cell proliferation and immune response processes, and thus play critical roles in the CD19-CAR-T therapy on B-ALL.

To further examine the functions of lncRNAs in these modules, we selected the top 200 gene–lncRNA pairs of high correlation (*R* > 0.9 and *P* < 1E−6) (Table S5). The lncRNAs that are highly correlated with histone and TF genes in our modules may be involved in the immune response/osteoclast differentiation/FOXO signaling (Figure S4C and Figure 4). Although the majority of highly co-expressed lncRNAs lack functional annotation, most of them may play important roles in the immune system. For example, the lncRNA *RN7SL1* in the turquoise module, which was reported to be associated with immune processes [25], displayed a high degree of co-expression with histone genes including *HIST1H4B* and *HIST1H2BL* (Figure 4).

**Figure 5:**
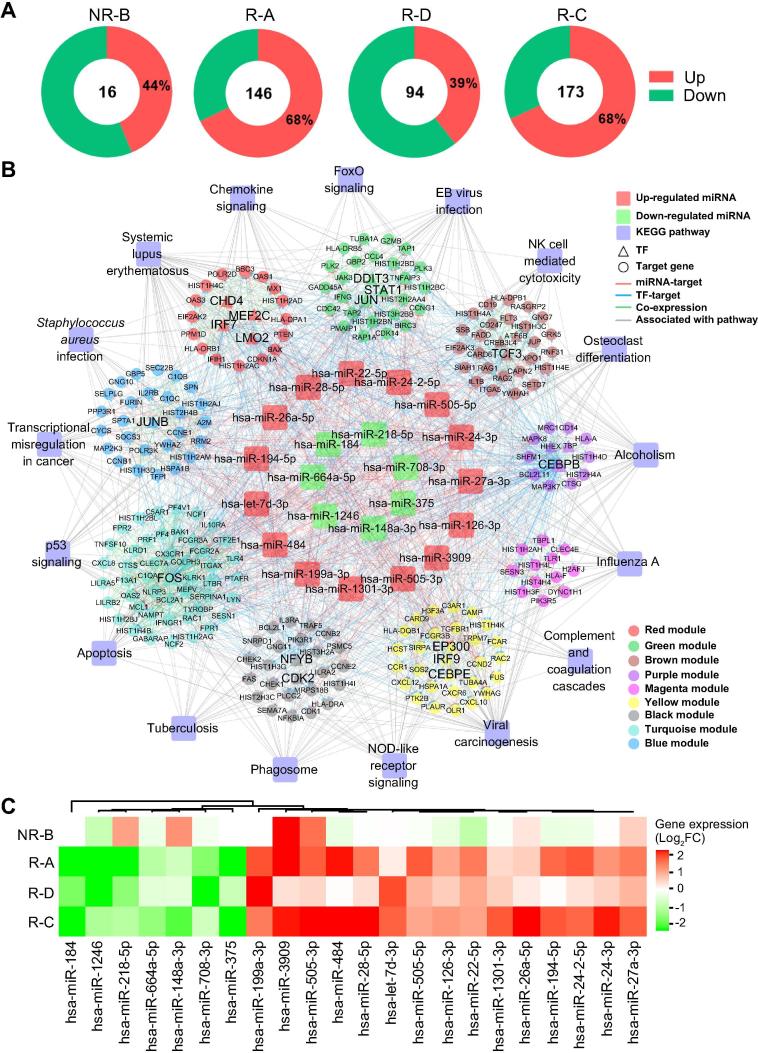
**The miRNA–TF–gene regulatory network involved in the CAR-T therapy** **A.** The distribution of DEMs across the four patients. The number of total DEGs and percentage of the up-regulated ones are provided. **B.** Important miRNA–TF–gene regulatory network. DEMs with a similar expression tendency in remissive patients were defined as important miRNAs, which together with TFs and their target genes formed the important miRNA-TF-gene regulatory network. The outer circle represents the KEGG pathways, the second circle represents the 9 co-expression modules depicted in different colors as shown in the legend box. The inner circle displays the 15 up-regulated miRNAs (red) and 7 down-regulated miRNAs (green) in the remissive samples. TFs and their target gene(s) are indicated in triangles and circles, respectively. Edges indicating miRNA–target regulation, TF–target regulation, co-expression between genes from different modules, and links between genes and pathways are shown in red, blue, green, and gray, respectively. **C.** Heatmap for DEMs with the same expression tendency in remissive patients. The color gradient from green to red indicates the fold change (log_2_ of post-*VS*-pre-CD19-CAR-T) of expression from low to high. DEM, differentially expressed miRNA.

Meanwhile, the expression levels of 16–173 miRNAs were significantly changed after CD19-CAR-T therapy (Figure 5A and Table S1), which were named as differentially expressed miRNAs (DEMs). The patient R-C with the best clinical outcome (Table 1) had the largest number of DEMs, while the patient NR-B had the least, suggesting important post-transcriptional roles of miRNAs involved in the CD19-CAR-T therapy on B-ALL. To further investigate how DEMs participating in the CD19-CAR-T therapy on B-ALL, we constructed a miRNA–TF–gene network to uncover potential regulatory modules (Figure 5B). This network contained 22 DEMs with a similar expression tendency in the remissive patients (Figure 5C). The 22 DEMs regulated the expression of 208 genes (20 TFs) in 9 functional modules, and a set of genes acted as key nodes crosslinking various pathways related to the immune system and cell cycle (Figure 5B). For instance, genes encoding TFs FOS, JUN, and CEBPB acted as crosstalk nodes in the biological processes related to apoptosis and the development of ALL (Figure 5B). The three TFs could regulate the expression of *HIST1H4A* and *HIST2H4A* [26], [27], which were targeted by miR-148a-3p in our network, suggesting that this regulatory loop may have an important role in the CD19-CAR-T therapy on B-ALL (Figure 5B). In addition, miRNA-375, whose expression was down-regulated in the remissive patients (Figure 5C), may regulate the expression of genes encoding TFs CHD4 and JUN, as well as HIST1H4C, that are involved in the NOD-like receptor signaling in our network (Figure 5B). Expression of miR-27a-3p, a tumor suppressor in B-ALL cell lines [28], was up-regulated after CAR-T therapy in samples from all four patients on D14 (Figure 5C). In our network, miR-27a-3p potentially regulates expression of a set of crosstalk genes (*e.g.*, *CEBPE* and *CHD4*) and participates in the immune response pathways (Figure 5B).

## Discussion

Previous clinical trials have reported that CAR-T therapy displayed dramatic efficacy in patients with B-ALL and non-Hodgkin's lymphoma [29]. In this study, we investigated the transcriptome profiling and regulatory networks of four B-ALL patients with different prognoses after CD19-CAR-T therapy. The co-expression and mRNA–miRNA regulatory network were constructed in an effort to identify potential functional modules underlying the CD19-CAR-T therapy on B-ALL. To the best of our knowledge, this is the first study to investigate the transcriptome profiling and regulatory mechanisms involved in CD19-CAR-T therapy.

Impressive results have been reported using CD19-CAR-T cells to treat patients with refractory B-ALL [4], [15], [16]. Our results are consistent with these reports that the malignant cells were eliminated, and 3 of 4 patients have achieved CR after CAR-T therapy (Figure S3D and Table 1). In addition, our findings demonstrate that the effect of CD19-CAR-T therapy on B-ALL is positively related to the abundance of CARs and the proportion of immune cell types in the BM (Figure 2B). In our trial, although *ex vivo* CAR-T cells comprised random T cell subtypes, the absence of NK and CD8^+^ T cells in the NR patient (Figure 2B) may be associated with the poor outcome and the low expression level of markers for functional CD8^+^ T cells (*PRF1*, *GZMA*, *GZMB*, *etc*.). Furthermore, the expression levels of tumor microenvironment related genes were dramatically changed after CAR-T infusion, such as immunostimulator/IL family/IFNR/GFR/chemokine families members, which may enhance the proliferation and activation of CAR-T cells and thus increase the anti-tumor activity [30].

Despite the differences in transcriptome profiles among these patients, most of the enriched DEG terms are related to the immune response and cell cycle (Figure 3). Our data demonstrate that histone family members were jointly and dynamically implicated and widely distributed in different functional modules associated with immune processes (Figure S4C and Figure 4), indicating their important roles in the CAR-T therapy on B-ALL. Moreover, our data show that histone and TF genes are strongly connected with most lncRNAs in the regulatory networks, suggesting the possible involvement of these lncRNAs in the CD19-CAR-T therapy (Figure S4). Functional relationships in the miRNA–TF–histone regulatory loop may play an essential role in CAR-T therapy. For example, miR-148a-3p, miR-27a-3p, and miR-375, which function as oncogenes or tumor suppressors, can also regulate the expression of hub TF genes *JUN* or *CEBPB* and histone genes *HIST1H4A*, *HIST1H4C*, and *HIST1H4E* [26], [27], [28], [31]. Expression of *HIST1H4A* and *HIST2H4A* is regulated by the TFs JUN and CEBPB as well [32], [33]. In our study, these TFs were co-expressed with the highest number of lncRNAs including lncRNA *HCG4P7*. *HCG4P7* is reportedly as an important immune regulatory molecule [34] and highly co-expressed with the gene encoding leukemia regulator *FOS* [35]. These networks could provide a valuable resource for investigating the transcriptional regulatory relationships involved in the effect of CD19-CAR-T therapy on B-ALL.

Although biological replications are limited due to the restrictions of medical ethics, previous studies have shown that the cancer cells of leukemia are homogeneously dispersed in the BM compared with the solid cancers [36]. Meanwhile, given the different genetic background of patients, the convergent results obtained from transcriptional profiling of the different patients could only partially explain the mechanisms underlying the processes of CAR-T therapy. In this study, the transcriptional profiling of BM from patients was performed using bulk RNA-seq, and alterations of the composition of cell types and their transcriptome profiles within the BM may provide valuable insights into the biological processes underlying CAR-T therapy. Although the alterations of important immune cell compositions were surveyed via bioinformatics approaches (Figure 2B), other types of cells in the BM, such as stromal and hematopoietic cells, have not been investigated.

Genetically-modified CAR-T cells act as “living drugs” to enable constant cytotoxic attacks on targeted malignant cells. The efficacy of CAR-T therapy depends on the tumor-specific antigens and further *in vivo* expansion of CAR-T cells [37]. Our results have shown the impact of *in vivo* expansion of CAR-T cells and the resulting alterations in immune cell population of CD19-CAR-T therapy on B-ALL. These could help to characterize clinically important features and develop treatments for patients with different conditions. Furthermore, our study suggests that the histone genes combined with their co-expressed lncRNAs and TFs, as well as the miRNA–TF–gene regulatory networks, may play vital roles in CD19-CAR-T therapy on B-ALL. These findings indicate an impact of these factors or modules for the CD19-CAR-T therapy on B-ALL, and may provide valuable clues for understanding the transcriptional and post-transcriptional regulatory mechanisms underlying CAR-T immunotherapy on cancers.

## Materials and methods

### Patient enrollment

All procedures in this trial, including sample collection, processing, freezing, and laboratory analysis *etc.*, were performed according to established standard operating procedures and protocols in the central laboratory at the Wuhan Union Hospital, China. Huazhong University of Science and Technology and the Wuhan Union Hospital ethics committees reviewed and approved this trial. All patients enrolled and treated in this trial gave written informed consent before participation. All clinical investigations were consistent with the Declaration of Helsinki. Only patients with relapsed or refractory B-ALL after standard therapies were deemed eligible for the CD19-CAR-T therapy.

### Preparation and fusion of CD19 CAR-T cells

The CD19-CAR transgene comprises five parts: CD19 single-chain variable fragment, CD8 hinge, CD8-α transmembrane, 4-1BB costimulatory domain, and CD3 zeta chain (Figure S1). The transgene was constructed into the lentiviral vector as shown in Figure S1, and then transferred into the donor T cells according to the protocols of Wuhan Sian Medical Technology (Wuhan, China). Briefly, the leukocytes were separated from the patient’s blood with the remainder of the blood returned to the patient’s circulation. Subsequently, the leukocytes were incubated with the lentiviral vector encoding the CAR for 10 days (Figure 1) according to the protocol [38].

To improve the efficacy of CAR-T therapy, patients were pre-treated with a conditioning chemotherapy agent (30 mg/m^2^ fludarabine and 750 mg/m^2^ cetuximab) for 5 days to control the MRD level below 20%. Afterward, patients received a fractionated infusion of CD19-CAR-T cells (1/3 dose at D1 and 2/3 dose at D2, respectively).

### Clinical and biomedical examinations

The PB and BM samples were obtained from patients on D0, D14, and D30. The percentage of CAR-T cells and normal cells in the CAR-T cell culture after *ex vivo* expansion (D0-EV) and in the PB and BM samples collected from patients on D14 were determined with flow cytometry. The MRD level in BM samples was measured using flow cytometry (FACSAria II, BD Pharmingen, San Diego, CA) on D0 and D30. Patients with the MRD level <0.5% after 2 weeks were considered as CR. The presence of *BCR-ABL* fusion transcript in BM samples was detected with a real-time PCR system (StepOnePlus, Applied Biosystems) with the primers (F: 5′-ACATCACGCCAGTCAACAG-3′ and R: 5′-GACGTAGAGCTTGCCATCAGA-3′). The tumor burden (TB) was calculated as the percentage of tumor cells among all karyocytes in BM samples on D0. Concentrations of cytokines in PB samples were determined with ELISA and with CRS grades (1–4) evaluated accordingly.

### RNA sequencing

Total RNA was isolated from the BM samples of all four patients on D0 and D14 using the standard TRIzol protocol. The RNA quality was determined with the Agilent 2100 Bioanalyzer. Libraries for RNA-seq (Ribo-Zero) and small RNA sequencing were prepared according to Illumina’s TruSeq protocol. The libraries were sequenced on the Illumina Hiseq platform with the 2 × 150 bp paired-end strategy at BGI-Shenzhen (Wuhan, China). Base-calling was performed using the Illumina CASAVA v1.8.2 pipeline.

RNA-seq reads containing <35 bp after adapter trimming or with poly-N or many low-quality bases (quality score ≤5 and the ratio of low-quality bases >10%) were removed. For small RNA sequencing reads, we filtered reads containing any N base or with a length >40 nt or <17 nt. The Q20, Q30, and GC content of the clean sequencing reads were calculated. All of the downstream analyses were based on the clean and high-quality sequencing reads.

### Bioinformatics analyses

Sequencing data obtained from the BM at day 0 and 14 days after CAR-T infusion were analyzed using various bioinformatics tools. The detailed procedures of transcriptome profiling, such as gene/miRNAs expression analysis, immune cell proportion estimation, functional enrichment analysis, and co-expression regulatory network analysis are presented in File S1.

## Data availability

Sequencing data in this study have been deposited in the Genome Sequence Archive [39] at the BIG Data Center [40], Beijing Institute of Genomics (BIG), Chinese Academy of Sciences, as GSA: CRA000746, which is publicly accessible at http://bigd.big.ac.cn/gsa.

## Authors’ contributions

AYG, QZ, YW, and JY conceived the project. AYG, QZ, and WY supervised the study. WY and JY performed the clinical trial and biochemical examinations. HH, QZ, SC, FH, and CL performed the bioinformatics analysis. HH and QZ drafted the manuscript with the help of WY. AYG, QZ, and HH revised the manuscript. All authors read and approved the final manuscript.

## Competing interests

The authors declare that they have no competing interests.

## Supplementary material

Supplementary File S1 **Detailed information about the bioinformatics analyses**

Supplementary Figure S1 **The schematic diagram of the CD19-CAR construction** The construct of 2nd generation CD19-CAR fragment is shown in the top right panel. The CD19-CAR fragment was cloned into the pSIAN lentiviral backbone and named as pSIAN-19-BB-z. The lentiviral vector was transferred to donor T cells from patients.

Supplementary Figure S2 **Gene Ontology and KEGG pathway enrichment analysis for the common highly-expressed genes (FPKM >100) in all samples** The color gradient varying from light blue to dark blue in the legend indicates the number of genes in each term from low to high. SRP-DCPTM, SRP-dependent cotranslational protein targeting to the membrane; BP, biological process; CC, cellular component; MF, molecular function.

Supplementary Figure S3 **Transcriptome profiling of BM samples from the 4 patients before and after CAR-T infusion A.** The violin plot shows the expression levels (log10 scale) of 10,263 genes (FPKM) and 470 miRNAs (TPM) in all BM samples on D0 and D14. The shape of the violin curve represents the distribution of the expression values. **B.** The common and specific highly expressed miRNAs (accounting for 70% of total expression for all miRNAs) in the BM samples from the four patients on D0 (left) and D14 (right), respectively. The inner circle indicates the common miRNAs while the petal indicates the ones specific to each patient. **C.** Score plot of the PLS-DA analysis showing the difference among samples. PC1 shows 30.7% of the variance, whereas PC2 shows 22.6% of the variance. The top 20 important genes ranked by the VIP score are shown on the left, and the heatmap showing expression levels (log2 scaled) of these genes among different samples is presented on the right. D. The heatmap showing the abundance of CDR3 among the different samples with the respective CDR3 sequences on the right. The color gradient from gray to dark red indicates the relative abundance of CDR3 from low to high. PC, principal component; FPKM, fragments per kilobase of transcript per million mapped reads; TPM, transcripts per million; PLS-DA, partial least squares discriminant analysis; VIP, variable importance in projection; CDR, complementarity-determining region.

Supplementary Figure S4 **Functional modules for DEGs identified using WGCNA A.** Eighteen co-expression modules identified using WGCNA. Modules were labeled in different colors, and the network heatmap displays the network overlap level of gene pairs in the corresponding modules. Red color indicates a high level of network overlap in the heatmap. **B.** KEGG pathway enrichment of the different co-expression modules. The module name and the corresponding pathways are shown on the Y axis, while the X axis shows the corresponding P values (−log10) for each enrichment. **C.** TF–gene–lncRNA co-expression regulatory network of the 9 modules indicated in B. Red, green, and gray nodes represent the genes with up-regulated expression, down-regulated expression, and expression patterns not sure, respectively, in the network of the 4 patients. lncRNAs are indicated with blue arrowheads and KEGG pathways are indicated with purple quadrangles. Edges indicating TF–target regulation, co-expression, and links between genes and pathways are shown in blue, green, and gray, respectively.
