# Supplementary material for: Transcriptome and Regulatory Network Analyses of CD19-CAR-T Immunotherapy for B-ALL
Source: Genomics Proteomics Bioinformatics. 2019 Jun 13;17(2):190–200. doi: 10.1016/j.gpb.2018.12.008 (PMC6620363; doi:10.1016/j.gpb.2018.12.008)
Supplement: Supplementary File S1 — Detailed information about the bioinformatics analyses [file mmc1.docx]

**File S1 Detailed information about the bioinformatics analyses**

## Estimation of gene/miRNA abundance, differential expression analysis, and clustering analysis

Clean reads from the miRNA-seq were mapped to canonical pre-miRNA human sequences from miRBase V21 to identify known miRNAs and estimate their expression profiles. The unmapped reads were aligned to GenBank, Rfam, and Piwi to identify rRNA, tRNA, snRNA, snoRNA, and piRNA. The normalized values (presented as transcripts per million reads; TPM) and read counts of the miRNAs in the different samples were merged into the matrix. The NOISeq [1] was used to assess the differentially expressed miRNAs (DEMs) with the significance threshold (probability > 0.9 and |fold change| > 2).

Transcript reassembly and quantification were processed according to the HISAT2-StringTie-ballgown pipeline [2] with Ensembl v83 (for protein-coding genes) and NONCODE v6 (for lncRNAs) annotations [3]. The resulting transcripts were pooled across samples using the merge function of StringTie [4], discarding all the redundant isoforms. The abundance of genes was estimated by StringTie and enumerated using FPKM (fragments per kilobase of transcript per million mapped reads). Genes with FPKM > 1 were kept for further analysis. Differentially expressed gene (DEG) analysis was performed using NOISeq with the significance threshold (probability > 0.9 and |fold change| > 2).

Partial-least squares discriminant analysis (PLS-DA) was employed to screen genes which could distinguish the non-remissive and remissive patients. The top ranked 20 genes with the highest variable importance in projection (VIP) score were selected to show their expression levels on D0 and D 14 (Figure S3C).

**Estimation of immune cell proportions and abundance of expressed CAR**

The surface marker gene lists of pro-B and pre-pro-B cells were curated from a previous report [5], and genes involved in the cytotoxic function of CD8^+^ T cells were collected from Samji et al ’s study [6]. The microenvironment related genes were collected from literature [7–9]. The distribution of immune cell populations and B-ALL malignant cells were estimated using EPIC [10], which could enumerate immune cell types from gene expression profiles of bulk RNA-Seq data. The abundance of CAR-T cells was estimated at the DNA and transcriptional levels: (1) flow cytometry and qPCR were employed to detect the abundance of CD19-CAR at the DNA level; and (2) the transcriptional level was estimated using the average depth of the CAR sequence. RNA-seq reads were aligned to reference CAR sequence, and the reads with a best match flag (*i.e.*, non-mismatch, non-multiple hit, and high quality) were kept to estimate the abundance of transcribed CAR sequences. FPKM was employed to normalize the abundance of CAR for a better comparison with other genes.

**Construction of co-expression modules and miRNA–TF–gene regulatory network**

The co-expression module construction and analysis were comprised of two steps. In the first step, we constructed weighted correlation co-expression networks for protein-coding genes using weighted correlation network analysis (WGCNA). We discarded the non-DEGs and genes with a low expression (FPKM < 1) in all samples, which may not contribute to the functional module detection. The soft-thresholding powers of gene co-expression networks (GCNs) were chosen using the criterion of scale-free topology [11] with an R^2 cut-off (0.9). Each branch of the hierarchical clustering dendrogram was considered to be a potential functional module in the network. To obtain modules with proper biological functions, we applied the dynamicTreeCut package [12] to cut trees, setting the minimum module size to 30 genes and the minimum height for merging modules at 0.25. Genes with the parameter kME > 0.7 were assigned to a module.

In the second step, we constructed the mRNA**–**lncRNA co-expression network based on results of the first step. (1) We built a core gene list that contains the differentially expressed lncRNAs and genes in the modules constructed in the first step, then used the gene list as an input matrix to construct GCNs again. (2) Given our interests in finding all possible mRNA**–**lncRNA co-expression modules, we set the smaller minimum module size as 30 and the merge cut height as 0.25, and the R^2 cut-off for soft-thresholding powers selection as 0.9. (3) We utilized the “democratic vote method” to assign genes to modules. Genes were assigned to a module only when their kME values are > 0.8. WGCNA network visualization was performed using VisANT software [13].

**Functional enrichment and co-expression regulatory network analyses**

Functional enrichment analysis was performed based on the hypergeometric test using in-house R scripts, terms with *P* < 0.01 were considered as enriched. The detailed method of miRNA**–**TF**–**gene regulatory network analysis was described in our previous work [14], and network visualization was performed using Cytoscape [15]. The TF gene list was downloaded from AnimalTFDB 2.0 [16]. Gene-based pathway crosstalk analysis was performed using ClueGO [17]. Other visualizations were implemented using R language (version 3.1.2) and its packages, such as gglot2 and heatmap. The method for co-expression analysis was similar with Ghanbarian and Hurst’ s study [18].

**References**

[1] Tarazona S, García F, Ferrer A, Dopazo J, Conesa A. NOIseq: a RNA-seq differential expression method robust for sequencing depth biases. EMBnetJournal 2011;17:18–9.

[2] Pertea M, Kim D, Pertea GM, Leek JT, Salzberg SL. Transcript-level expression analysis of RNA-seq experiments with HISAT, StringTie and Ballgown. Nat Protoc 2016;11:1650–67.

[3] Zhao Y, Li H, Fang S, Kang Y, wu W, Hao Y, et al. NONCODE 2016: an informative and valuable data source of long non-coding RNAs. Nucleic Acids Res 2016;44:D203–8.

[4] Pertea M, Pertea GM, Antonescu CM, Chang TC, Mendell JT, Salzberg SL. StringTie enables improved reconstruction of a transcriptome from RNA-seq reads. Nat Biotechnol 2015;33:290–5.

[5] Tokoyoda K, Egawa T, Sugiyama T, Choi BI, Nagasawa T. Cellular niches controlling B lymphocyte behavior within bone marrow during development. Immunity 2004;20:707–18.

[6] Samji T, Khanna KM. Understanding memory CD8+ T cells. Immunol Lett 2017;185:32–9.

[7] Mbeunkui F, Johann DJ. Cancer and the tumor microenvironment: a review of an essential relationship. Cancer Chemother Pharmacol 2009;63:571–82.

[8] Korneev KV, Atretkhany KSN, Drutskaya MS, Grivennikov SI, Kuprash DV, Nedospasov SA. TLR-signaling and proinflammatory cytokines as drivers of tumorigenesis. Cytokine 2017;89:127–35.

[9] Kessenbrock K, Plaks V, Werb Z. Matrix metalloproteinases: regulators of the tumor microenvironment. Cell 2010;141:52–67.

[10] Racle J, de Jonge K, Baumgaertner P, Speiser DE, Gfeller D. Simultaneous enumeration of cancer and immune cell types from bulk tumor gene expression data. Elife 2017;6.

[11] Zhang B, Horvath S. A general framework for weighted gene co-expression network analysis. Stat Appl Genet Mol Biol 2005;4:Article17.

[12] Langfelder P, Zhang B, Horvath S. Defining clusters from a hierarchical cluster tree: the dynamic tree cut package for R. Bioinformatics 2008;24:719–20.

[13] Hu Z, Mellor J, Wu J, Yamada T, Holloway D, DeLisi C. VisANT: data-integrating visual framework for biological networks and modules. Nucleic Acids Res 2005;33:W352–7.

[14] Zhang HM, Kuang S, Xiong X, Gao T, Liu C, Guo AY. Transcription factor and microRNA co-regulatory loops: important regulatory motifs in biological processes and diseases. Brief Bioinform 2015;16:45–58.

[15] Shannon P, Markiel A, Ozier O, Baliga NS, Wang JT, Ramage D, et al. Cytoscape: a software environment for integrated models of biomolecular interaction networks. Genome Res 2003;13:2498–504.

[16] Zhang HM, Liu T, Liu CJ, Song S, Zhang X, Liu W, et al. AnimalTFDB 2.0: a resource for expression, prediction and functional study of animal transcription factors. Nucleic Acids Res 2015;43:D76–81.

[17] Bindea G, Mlecnik B, Hackl H, Charoentong P, Tosolini M, Kirilovsky A, et al. ClueGO: a Cytoscape plug-in to decipher functionally grouped gene ontology and pathway annotation networks. Bioinforma Oxf Engl 2009;25:1091–3.

[18] Ghanbarian AT, Hurst LD. Neighboring genes show correlated evolution in gene expression. Mol Biol Evol 2015;32:1748–66.
