# Supplementary material for: Transcriptome and Regulatory Network Analyses of CD19-CAR-T Immunotherapy for B-ALL
Source: Genomics Proteomics Bioinformatics. 2019 Jun 13;17(2):190–200. doi: 10.1016/j.gpb.2018.12.008 (PMC6620363; doi:10.1016/j.gpb.2018.12.008)
Supplement: Supplementary Figure S1 — The schematic diagram of the CD19-CAR construction The construct of 2nd generation CD19-CAR fragment is shown in the top right panel. The CD19-CAR fragment was cloned into the pSIAN lentiviral backbone and named as pSIAN-19-BB-z. The lentiviral vector was transferred to donor T cells from patients. [file mmc2.pdf]

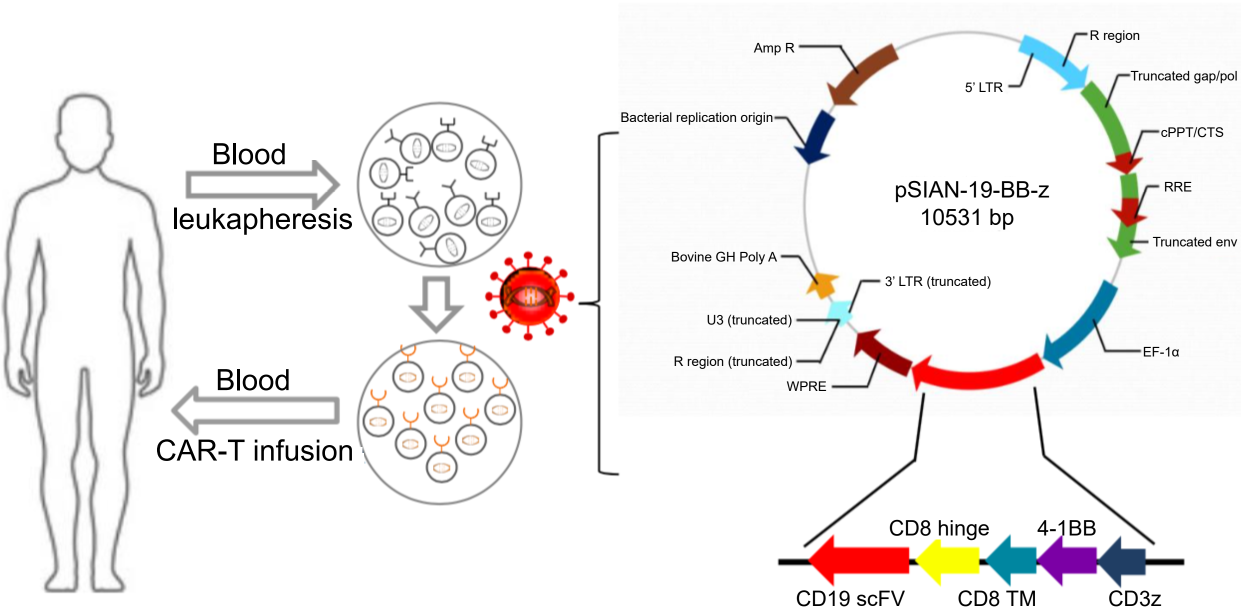
Blood
leukapheresis
Blood
CAR-T infusion
Amp R
R region
5' LTR
Truncated gap/pol
Bacterial replication origin
cPPT/CTS
pSIAN-19-BB-z
10531 bp
RRE
Truncated env
Bovine GH Poly A
3' LTR (truncated)
U3 (truncated)
R region (truncated)
WPRE
EF-1α
CD8 hinge
4-1BB
CD19 scFV
CD8 TM
CD3z
