# Supplementary figures and images for: Transcriptome and Regulatory Network Analyses of CD19-CAR-T Immunotherapy for B-ALL
Source: Genomics Proteomics Bioinformatics. 2019 Jun 13;17(2):190–200. doi: 10.1016/j.gpb.2018.12.008 (PMC6620363; doi:10.1016/j.gpb.2018.12.008)

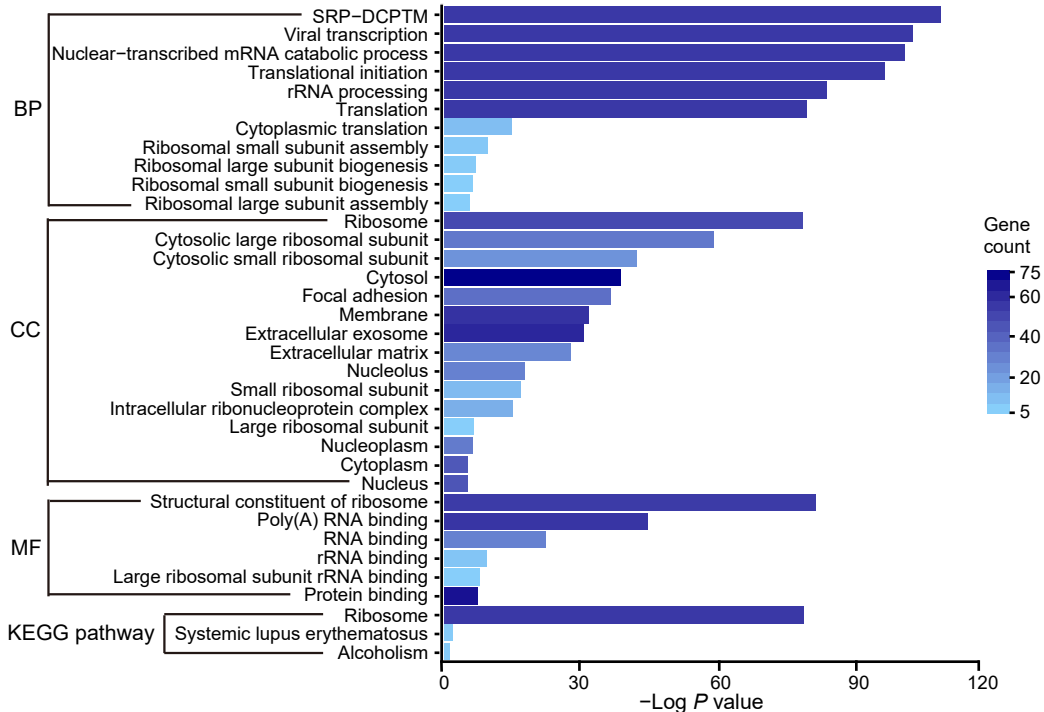

Supplement: Supplementary Figure S2 — Gene Ontology and KEGG pathway enrichment analysis for the common highly-expressed genes (FPKM >100) in all samples The color gradient varying from light blue to dark blue in the legend indicates the number of genes in each term from low to high. SRP-DCPTM, SRP-dependent cotranslational protein targeting to the membrane; BP, biological process; CC, cellular component; MF, molecular function. [file mmc3.pdf]

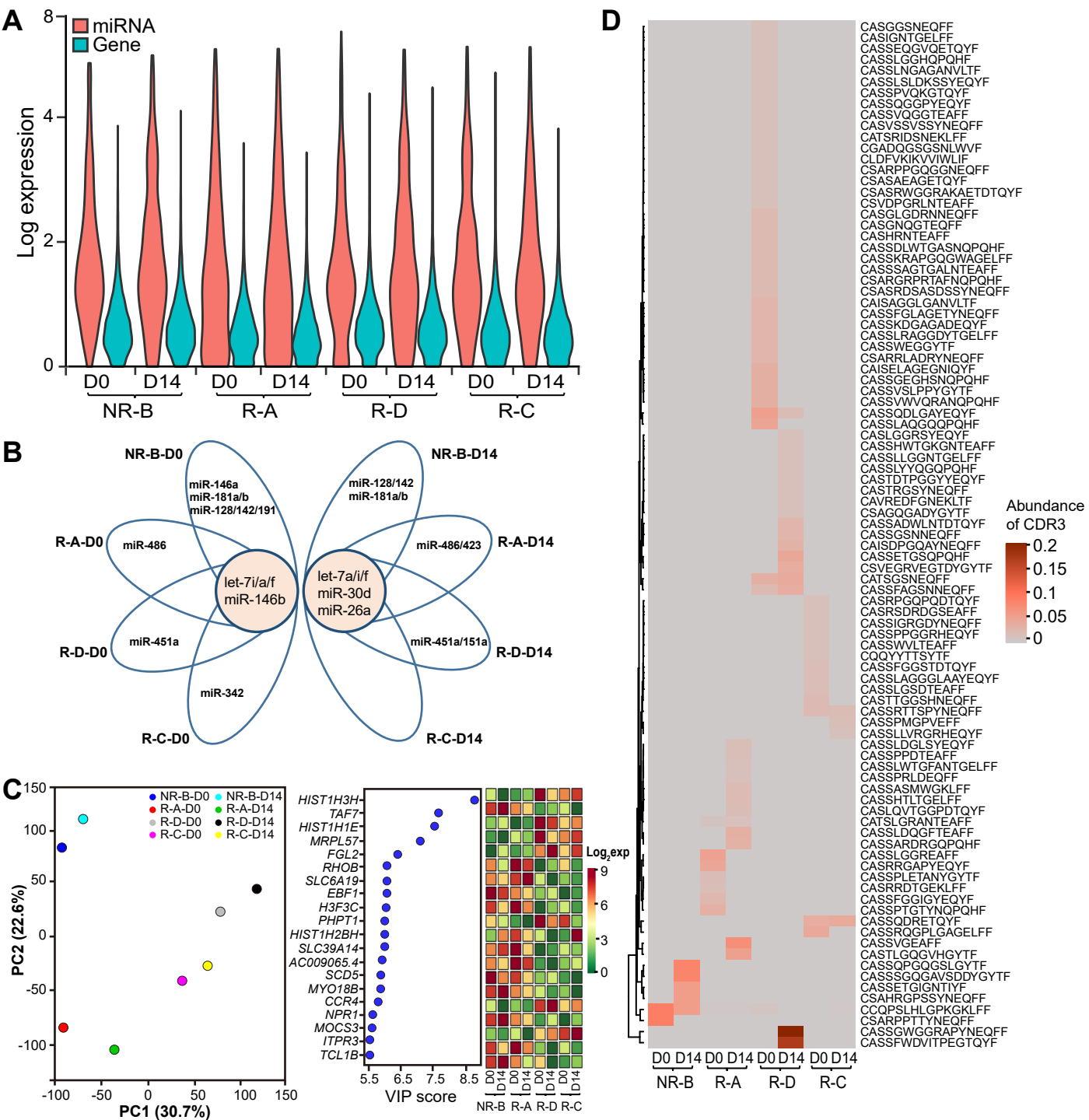

Supplement: Supplementary Figure S3 — Transcriptome profiling of BM samples from the 4 patients before and after CAR-T infusion A. The violin plot shows the expression levels (log10 scale) of 10,263 genes (FPKM) and 470 miRNAs (TPM) in all BM samples on D0 and D14. The shape of the violin curve represents the distribution of the expression values. B. The common and specific highly expressed miRNAs (accounting for 70% of total expression for all miRNAs) in the BM samples from the four patients on D0 (left) and D14 (right), respectively. The inner circle indicates the common miRNAs while the petal indicates the ones specific to each patient. C. Score plot of the PLS-DA analysis showing the difference among samples. PC1 shows 30.7% of the variance, whereas PC2 shows 22.6% of the variance. The top 20 important genes ranked by the VIP score are shown on the left, and the heatmap showing expression levels (log2 scaled) of these genes among different samples is presented on the right. D. The heatmap showing the abundance of CDR3 among the different samples with the respective CDR3 sequences on the right. The color gradient from gray to dark red indicates the relative abundance of CDR3 from low to high. PC, principal component; FPKM, fragments per kilobase of transcript per million mapped reads; TPM, transcripts per million; PLS-DA, partial least squares discriminant analysis; VIP, variable importance in projection; CDR, complementarity-determining region. [file mmc4.pdf]

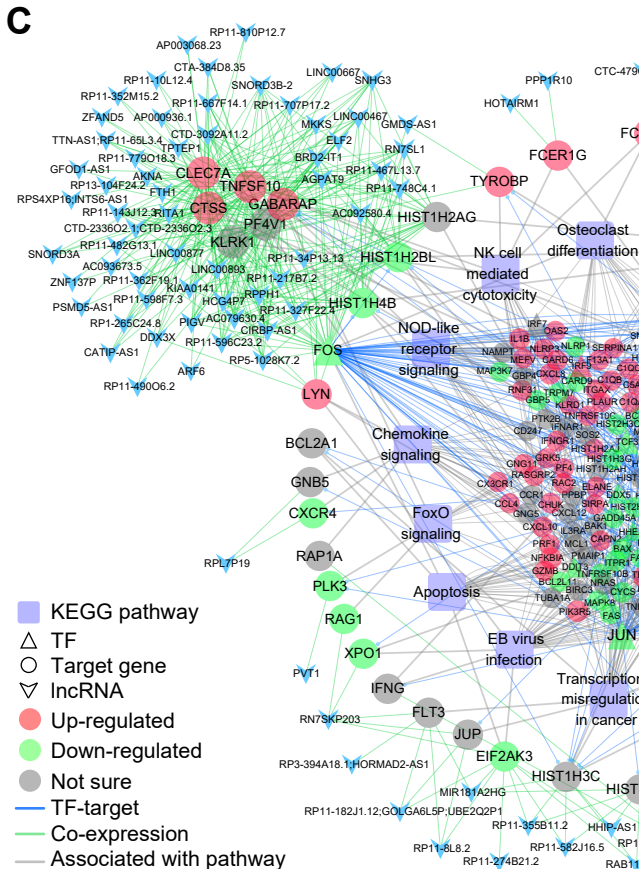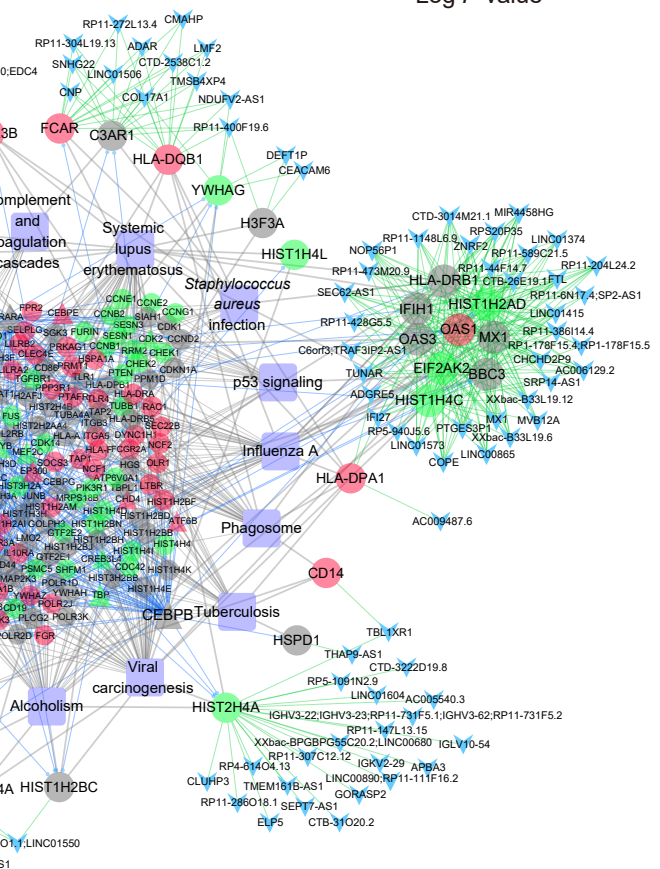

Supplement: Supplementary Figure S4 — Functional modules for DEGs identified using WGCNA A. Eighteen co-expression modules identified using WGCNA. Modules were labeled in different colors, and the network heatmap displays the network overlap level of gene pairs in the corresponding modules. Red color indicates a high level of network overlap in the heatmap. B. KEGG pathway enrichment of the different co-expression modules. The module name and the corresponding pathways are shown on the Y axis, while the X axis shows the corresponding P values (−log10) for each enrichment. C. TF–gene–lncRNA co-expression regulatory network of the 9 modules indicated in B. Red, green, and gray nodes represent the genes with up-regulated expression, down-regulated expression, and expression patterns not sure, respectively, in the network of the 4 patients. lncRNAs are indicated with blue arrowheads and KEGG pathways are indicated with purple quadrangles. Edges indicating TF–target regulation, co-expression, and links between genes and pathways are shown in blue, green, and gray, respectively. [file mmc5.pdf]
